# Assessment of the required performance and the development of corresponding program decision rules for neglected tropical diseases diagnostic tests: Monitoring and evaluation of soil-transmitted helminthiasis control programs as a case study

**DOI:** 10.1371/journal.pntd.0009740

**Published:** 2021-09-14

**Authors:** Bruno Levecke, Luc E. Coffeng, Christopher Hanna, Rachel L. Pullan, Katherine M. Gass

**Affiliations:** 1 Department of Virology, Parasitology, Immunology and Physiology, Ghent University, Merelbeke, Belgium; 2 Department of Public Health, Erasmus MC, University Medical Centre Rotterdam, Rotterdam, the Netherlands; 3 Global Project Partners, LLC, Oakland, California, United States of America; 4 Department of Disease Control, London School of Hygiene and Tropical Medicine, London, United Kingdom; 5 Neglected Tropical Diseases Support Centre, The Task Force for Global Health, Decatur, Georgia, United States of America; NIH-National Institute for Research in Tuberculosis-ICER, INDIA

## Abstract

Recently, the World Health Organization established the Diagnostic Technical Advisory Group to identify and prioritize diagnostic needs for neglected tropical diseases, and to ultimately describe the minimal and ideal characteristics for new diagnostic tests (the so-called target product profiles (TPPs)). We developed two generic frameworks: one to explore and determine the required sensitivity (probability to correctly detect diseased persons) and specificity (probability to correctly detect persons free of disease), and another one to determine the corresponding samples sizes and the decision rules based on a multi-category lot quality assurance sampling (MC-LQAS) approach that accounts for imperfect tests. We applied both frameworks for monitoring and evaluation of soil-transmitted helminthiasis control programs. Our study indicates that specificity rather than sensitivity will become more important when the program approaches the endgame of elimination and that the requirements for both parameters are inversely correlated, resulting in multiple combinations of sensitivity and specificity that allow for reliable decision making. The MC-LQAS framework highlighted that improving diagnostic performance results in a smaller sample size for the same level of program decision making. In other words, the additional costs per diagnostic tests with improved diagnostic performance may be compensated by lower operational costs in the field. Based on our results we proposed the required minimal and ideal diagnostic sensitivity and specificity for diagnostic tests applied in monitoring and evaluating of soil-transmitted helminthiasis control programs.

## Author summary

The World Health Organization established an advisory group to identify and prioritize diagnostic needs for neglected tropical diseases, and to ultimately describe the minimal and ideal characteristics for new diagnostic tests. To support this advisory group, we developed two generic frameworks, which we applied to soil-transmitted helminthiases: one to explore and determine the required sensitivity (probability to correctly detect a diseased person) and specificity (probability to correctly detect a person free of disease), and another one to determine the corresponding samples size and decision rules during surveys. We showed that specificity rather than sensitivity will become more important when the program approaches the endgame of elimination and that the requirements for both parameters are inversely correlated, resulting in multiple combinations of sensitivity and specificity that allow for reliable decision making. We also highlighted that improving diagnostic performance results in smaller sample sizes for the same level of program decision making. In other words, the additional costs per diagnostic tests with improved diagnostic performance can be compensated by the lower operational costs in the field. Based on our results we proposed to the advisory group the required performance characteristics of diagnostic tests for soil-transmitted helminthiasis control programs.

## Introduction

Recently, the Strategic and Technical Advisory Group (STAG), the principal advisory group to the World Health Organization (WHO) for the control of neglected tropical diseases (NTDs), decided that a single WHO working group was needed to help to identify and prioritize diagnostic needs [1]. One of the recommendations was that target product profiles (TPPs) for diagnostics were needed for soil-transmitted helminths (STHs) that would facilitate monitoring and evaluation of soil-transmitted helminthiasis control programs [2]. Generally, these TPPs describe the minimal and ideal characteristics, including but not limited to the sensitivity and the specificity (see [3] for previously published TPPs).

Soil-transmitted helminthiasis is a parasitic disease caused by a group of intestinal roundworms, including *Ascaris lumbricoides* (giant roundworm), *Trichuris trichiura* (whipworm), *Ancylostoma duodenale* and *Necator americanus* (hookworms). In 2019, it was estimated that they globally accounted for 1.97 million disability adjusted life years (12% of the total disease burden attributed to NTDs [4]). Given the route of STH transmission, infections and the associated disease burden predominantly occurs in (sub)tropical countries where transmission is facilitated by the optimal climate conditions for larval development, poverty, and lack of both sanitation and hygiene [4,5]. To fight the global STH-attributable morbidity, WHO recommends preventive chemotherapy (PC) programs, during which a single tablet of anthelmintic drugs (albendazole (400 mg) or mebendazole (500 mg)) is periodically administered to both pre-school and school age children and other at-risk populations living in endemic areas. The frequency of these large-scale deworming programs is based on whether the observed prevalence of STH infections (any species) exceeds a predefined program decision threshold. For example, at the start of the program it is recommended to distribute drugs twice a year when the prevalence is at least 50% and once a year when the prevalence is at least 20%. During the implementation phase, the prevalence of any STH infection is periodically re-evaluated to verify whether objectives are being met, and if necessary, to adjust the frequency of drug administration (prevalence ≥50%: 3x PC / year; 50%> prevalence ≥20%: maintain PC frequency; 20%> prevalence ≥10%: 1x PC /year; 10%> prevalence ≥2%: 1x PC/2 years; prevalence <2%: no PC) [6].

Traditionally, STHs have been diagnosed by detecting worm specific eggs in stool using a compound light microscope. Since the 1990s, Kato-Katz has been the WHO recommended diagnostic standard for quantifying eggs in stools [7], and hence it has been used to guide soil-transmitted helminthiasis control programs. During the last decade, a variety of new diagnostic tests have been introduced to the STH field, including both other microscopy-based [8–10], and DNA-based methods [11]. Each of these tests have important advantages and disadvantages over the Kato-Katz. Important advantages are a clearer microscopic view [8,9], a higher clinical sensitivity (referring to the proportion of diseased individuals correctly diagnosed as infected) [12,13], opportunities for automated egg counting and quality control [10,14], the ability to differentiate hookworm species [11] and to simultaneously detect parasites other than STHs [8,9,11]. The chief limitations of these novel tests are the need for well-equipped laboratories with well-trained technicians, the need to transport samples to a distant laboratory, the higher cost of processing large numbers of samples [15,16], and the lack of standardized protocols for DNA-based methods [11,17,18]. Currently, most diagnostic technologies based on biomarkers other than eggs or DNA (e.g. antigens, antibodies and metabolites) or other sample matrices (e.g. serum and urine) are either not yet explored or in research phase [19–22]. As these new diagnostic technologies transit from research to routine program tools, important consideration needs to be paid to the performance of these tools when used by NTD programs for making public health decisions.

In the present study, we developed a generic framework to explore the impact of diagnostic test sensitivity and specificity at the individual level on program decision making at the population level, with the ultimate aim to better define minimum TPP sensitivity and specificity targets for diagnostic tests for PC targeted NTDs. To this end, we first explored the impact of diagnostic sensitivity and specificity on the probability of making an incorrect program decision within a soil-transmitted helminthiasis control program: unnecessarily selecting a PC frequency that is greater than indicated by the true prevalence or prematurely reducing the frequency of PC. Subsequently, we developed a multi-category lot quality assurance sampling (MC-LQAS) framework that incorporates imperfect test performance to determine the corresponding sample size and associated decision rules.

## Methods

### Required sensitivity and specificity

#### General framework

A program decision is generally based on the outcome of an epidemiological survey in which *N*_*tot*_ subjects are screened for the presence of any infection. The observed prevalence (proportion of positive test results *N*^+^ out of *N*_*tot*_, which includes both false and true positive test results) is then compared to a program decision threshold (*T*). Rather than a proportion, one can also verify whether the number of positive test results *N*^+^ exceeds *T*′. When we assume a diagnostic test *D* with a sensitivity of *Se*_*d*_ and a specificity *Sp*_*d*_, a true underlying prevalence equal to *Prev*_*true*_ and a sample size of *N*_*tot*_, the probability observing at least *T*′ positive results can be written as

P(N+≥T′|Prevtrue,Sed,Spd,Ntot)=∑x=T′Ntot(Ntotx)∙Prob+x∙(1−Prob+)Ntot−x (1)

$${Prob}_{+}={Se}_{d}∙{Prev}_{true}+(1-{Sp}_{d})∙(1-{Prev}_{true})$$ (2)

It is important to note that *T*′ is not a fixed value, rather it will be a function of the total number of subjects screened (*N*_*tot*_), the program decision threshold (*T*) and the diagnostic performance of the test (*Se*_*d*_ and *Sp*_*d*_), and this can be best illustrated with a few toy examples. Assume that we are screening 500 subjects (*N*_*tot*_) with a perfect test (*Se*_*d*_ = *Sp*_*d*_ = 100%) and the program decision threshold *T* is set at 50%, then *T*′ equals 250. In case 1,000 subjects are screened with a perfect test, *T*′ equals 500. Given the same *N*_*tot*_ (1,000 subjects) and diagnostic performance but a *T* of 2% instead of 50%, *T*′ equals 20. When an imperfect diagnostic test (*Se*_*d*_ = 80% and *Sp*_*d*_ = 80%) is used to screen 1,000 subjects and decisions are made around a program decision threshold *T* of 2%, *T*′ equals 212 or more generally

$$T\prime =N_{tot}∙({Se}_{d}∙T+(1-{Sp}_{d})∙(1-T))$$ (3)

Combining **(1)–(3)** allows one to explore the impact of *Se*_*d*_ and *Sp*_*d*_ on the probability of making an incorrect program decision around a set of program decision thresholds *T*. For example, suppose 500 subjects (*N*_*tot*_) are randomly selected from a population where the true underlying prevalence equals 45% (*Prev*_*true*_) and a threshold of 50% (*T*) is used to make program decisions. The probability of *N*^+^ ≥ *T*′, and therefore unnecessarily selecting a PC frequency that is higher than indicated by the true prevalence, equals 1.4% when a perfect test (*Se*_*d*_ = *Sp*_*d*_ = 100%) is applied and 9.7% for an imperfect test (*Se*_*d*_ = *Sp*_*d*_ = 80%). Similarly, one can determine the probability of prematurely reducing the PC frequency. For example, if we change the true underlying prevalence from 45% to 55% (*Prev*_*true*_ ≥ *T*), the probability of *N*^+^ < *T*′, and therefore prematurely reducing the PC frequency equals 1.1% (= 1 – the probability of *N*^+^ ≤ *T*′) when a perfect test (*Se*_*d*_ = *Sp*_*d*_ = 100%) is applied and 8.2% for the same imperfect test (*Se*_*d*_ = *Sp*_*d*_ = 80%).

#### Data generation

For this analysis, we fixed *N*_*tot*_ to 500, but varied both *Se*_*d*_ and *Sp*_*d*_ from 60% to 100% with 1% increments (resulting in 41 x 41 theoretic diagnostic tests) and *Prev*_*true*_ from 0% to 100% with 0.2% increments. The program decision thresholds included the currently recommended thresholds for an STH control program (2%, 10%, 20% and 50%). In addition, we included program thresholds of 1% and 5%. This is because the current program thresholds are based on the observed prevalence using Kato-Katz thick smear, for which we know the specificity is not 100% [23,24]. As a consequence of this, the true underlying prevalence might be overestimated as it approaches zero.

#### Analysis of generated data

**Fig 1:**
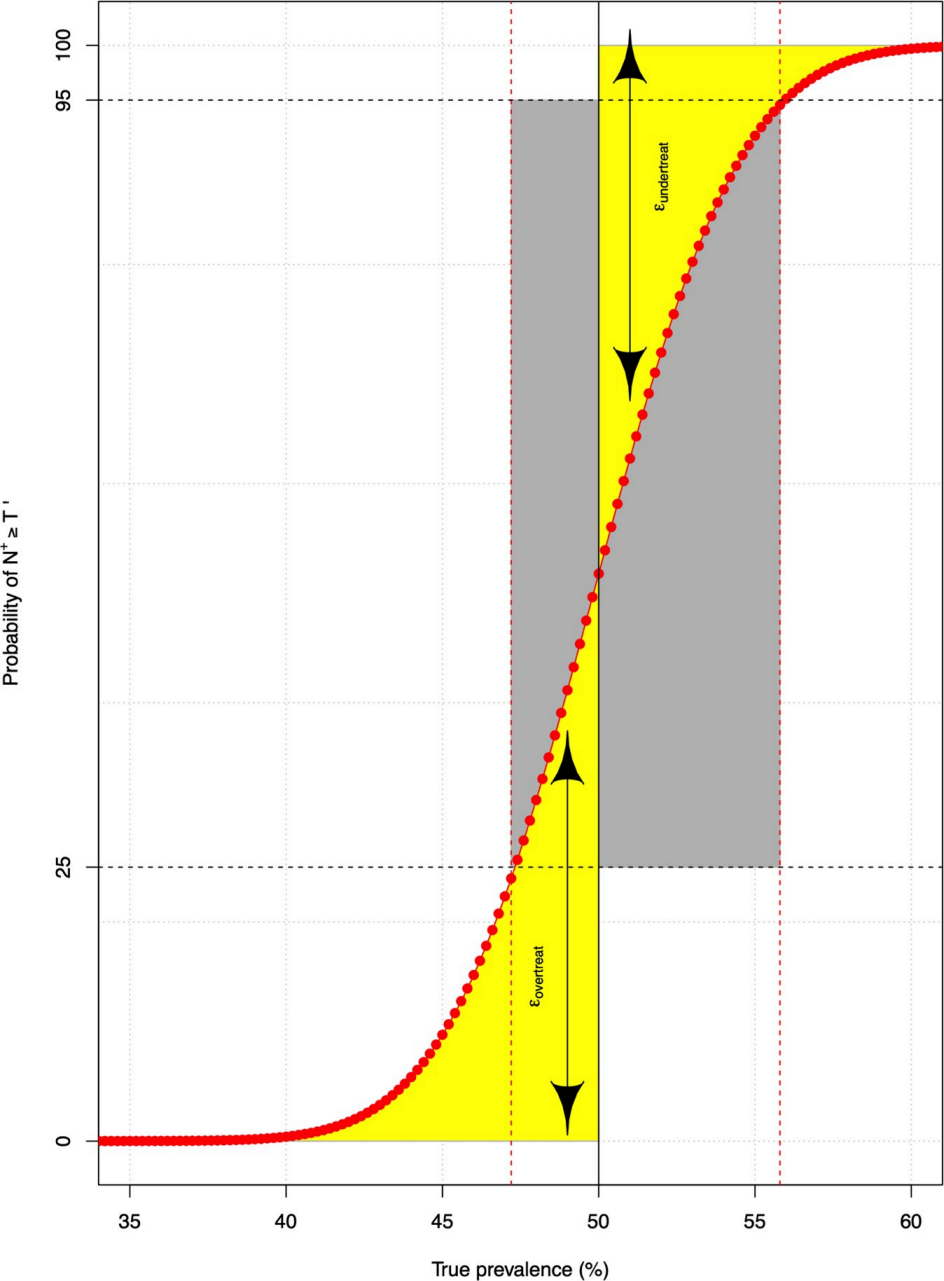
The general framework to determine the required sensitivity and specificity. The red line represents the probability (in %) of the number of positive test results (*N*^+^*)* in a random sample of *N*_*tot*_ subjects (= 500) being at least *T*′ (see **Eq 3**) based on an imperfect diagnostic test *D* (sensitivity (*Se*_*d*_) = specificity (*Sp*_*d*_) = 80%) over a wide range of true underlying prevalence (*Prev*_*true*_). The vertical straight line represents the program decision threshold *T* of 50%. The yellow areas highlight the program errors *ε*_*overtreat*_ (*Prev*_*true*_<50%) and *ε*_*undertreat*_ (*Prev*_*true*_≥50%). The horizontal black dashed lines represent a *ε*_*overtreat*_ equal to 25% and a *ε*_*undertreat*_ equal to 5% (= 100% - 95%), the vertical red dashed lines indicate the corresponding *Prev*_*true*_. The grey zone indicates the range of *Prev*_*true*_ for which the diagnostic test is considered inadequate to make a well-informed program decision (*ε*_*overtreat*_>25% and *ε*_*undertreat*_>50%).

To further illustrate the interpretation of the obtained data, we worked out a toy example in **Fig 1.** This figure represents the probability of *N*^+^ ≥ *T*′ over a wide range of *Prev*_*true*_ when an imperfect diagnostic test (*Se*_*d*_ = *Sp*_*d*_ = 80%) was applied. Given a program decision threshold *T* of 50% (vertical straight line), we can deduce both the error related to unnecessarily selecting a PC frequency that is greater than needed (*ε*_*overtreat*_) or prematurely reducing the frequency of PC (*ε*_*undertreat*_). These errors are analogous to 1 minus the negative predictive value and 1 minus the positive predicted value, as used in recent NTD modelling studies on optimal program decision thresholds [25–27]. Subsequently, we can also deduce to what extent this diagnostic test allows for reliable decision making. In the present study, we will use two different operating definitions for ‘reliable’ based on both errors. In both definitions, we set the highest allowed probability of prematurely reducing frequency (*E*_*undertreat*_) at 5%, whereas the highest allowed probability of falsely continuing or increasing PC frequency (*E*_*overtreat*_) was set at either 10% and 25%. Generally, a lower value for *E*_*undertreat*_ is preferred as prematurely reducing PC frequency may lead to an increase in infection and morbidity. The two values for *E*_*overtreat*_ allow to differentiate between both adequate (*E*_*overtreat*_ = 25%) and ideal (*E*_*overtreat*_ = 10%) program decision making scenarios. In the remainder of the document, we will refer to (in)adequate and (less than) ideal program decision making when the *E*_*overtreat*_ is set at 25% and 10% respectively. The values for *E*_*undertreat*_ and *E*_*overtreat*_ here have also been applied earlier to determine the sensitivity and specificity for diagnostic tests for other helminth diseases [28].

In the toy example (**Fig 1**), the diagnostic test performed at *ε*_*undertreat*_≤5% when *Prev*_*true*_ is at least 55.8% and at *ε*_*overtreat*_≤25% when the *Prev*_*true*_ is not higher than 47.2%. In other words, any program decision making within the *Prev*_*true*_ interval] 47.2; 55.8 [is considered inadequate when applying this test; we will refer to this interval as the ‘grey zone’. It is expected that for a given sample size, the grey zone narrows with higher levels of sensitivity and specificity of diagnostic methods. Because the width of grey zones also depends on binomial variation, and thus on the program decision threshold itself, we quantified the grey zone for each combination *Se*_*d*_ and *Sp*_*d*_ and program decision threshold separately.

In order to further differentiate diagnostic tests with small grey zones from those with a wider zone, we classified the grey zone into three levels (level 1–3) for each program decision threshold *T* separately. This classification into 3 levels was based on the 25^th^ and 75^th^ percentile of the width of the grey zones (level 1: width of grey zone < 25^th^ percentile; level 2: 75^th^ percentile > width of grey zone ≥ 25^th^ percentile; level 3: width of grey zone ≥ 75^th^ percentile (see **S1 Table**) across all potential diagnostic methods that allowed for adequate program decision making. In other words, each of these diagnostic methods allowed for adequate decision making (*E*_*overtreat*_ is set at 25%) at a true underlying prevalence of zero and 100%. Finally, we arbitrarily classified the diagnostic tests into ‘minimal’ and ‘optimal’ based on their corresponding levels of grey zone across each of the 6 program decisions thresholds. Diagnostic performance was considered optimal when they resulted in level 1 grey zone for at least 3 out of the 6 program decision thresholds and did not result in a level 3 grey zone in any of the 6 program thresholds. In all other cases, the diagnostic test was considered ‘minimal’.

### MC-LQAS framework

#### General framework for LQAS

Lot quality assurance sampling (LQAS) is a technique to gather the minimal amount of information required for decision making, using a sample size as small as possible. Instead of constructing a precise estimate of a population parameter, LQAS aims to quantify whether the population parameter is above or below some decision cut-off *c* with some desired minimal probability. For STH, LQAS can be used to verify whether the observed number of positive test results (*N*^+^) in a random sample (*N*_*tot*_) equals or exceeds a predefined decision cut-off *c* [29,30], followed by continuing the current PC frequency if this is the case, and reducing the PC frequency in all other cases. The sample size *N*_*tot*_ and the corresponding decision cut-off *c* are chosen to satisfy two conditions. The first is that for some prevalence *Prev*_*true*_ less than the program decision threshold *T* (*Prev*_*true*<*T*_), the probability *ε*_*overtreat*_ to select a PC frequency that is higher than indicated by the true underlying prevalence does not exceed the target probability *E*_*overtreat*_. The second condition is that for some *Prev*_*true*_ equal or above the program decision threshold *T* (*Prev*_*true*≥*T*_), the probability *ε*_*undertreat*_ to prematurely reduce the PC frequency is not higher than *E*_*undertreat*_. Based on Eqs **(1)–(3)** one can write these conditions as

P(N+≥c|Prevtrue<T,Sed,Spd,Ntot)=∑x=cNtot(Ntotx)∙Prob+x∙(1−Prob+)Ntot−x≤Eovertreat (4)

P(N+<c|Prevtrue≥T,Sed,Spd,Ntot)=∑x=0c−1(Ntotx)∙Prob+x∙(1−Prob+)Ntot−x≤Eundertreat (5)

where *Prob*_+_ equals *Se*_*d*_⋅*Prev*_*true*<*T*_+(1−*Sp*_*d*_)∙(1−*Prev*_*true*<*T*_) in **(4)** and *Se*_*d*_⋅*Prev*_*true*≥*T*_+(1−*Sp*_*d*_)∙(1−*Prev*_*true*≥*T*_) in **(5).**

#### Process to determine the decision cut-off *c* within LQAS

**Fig 2:**
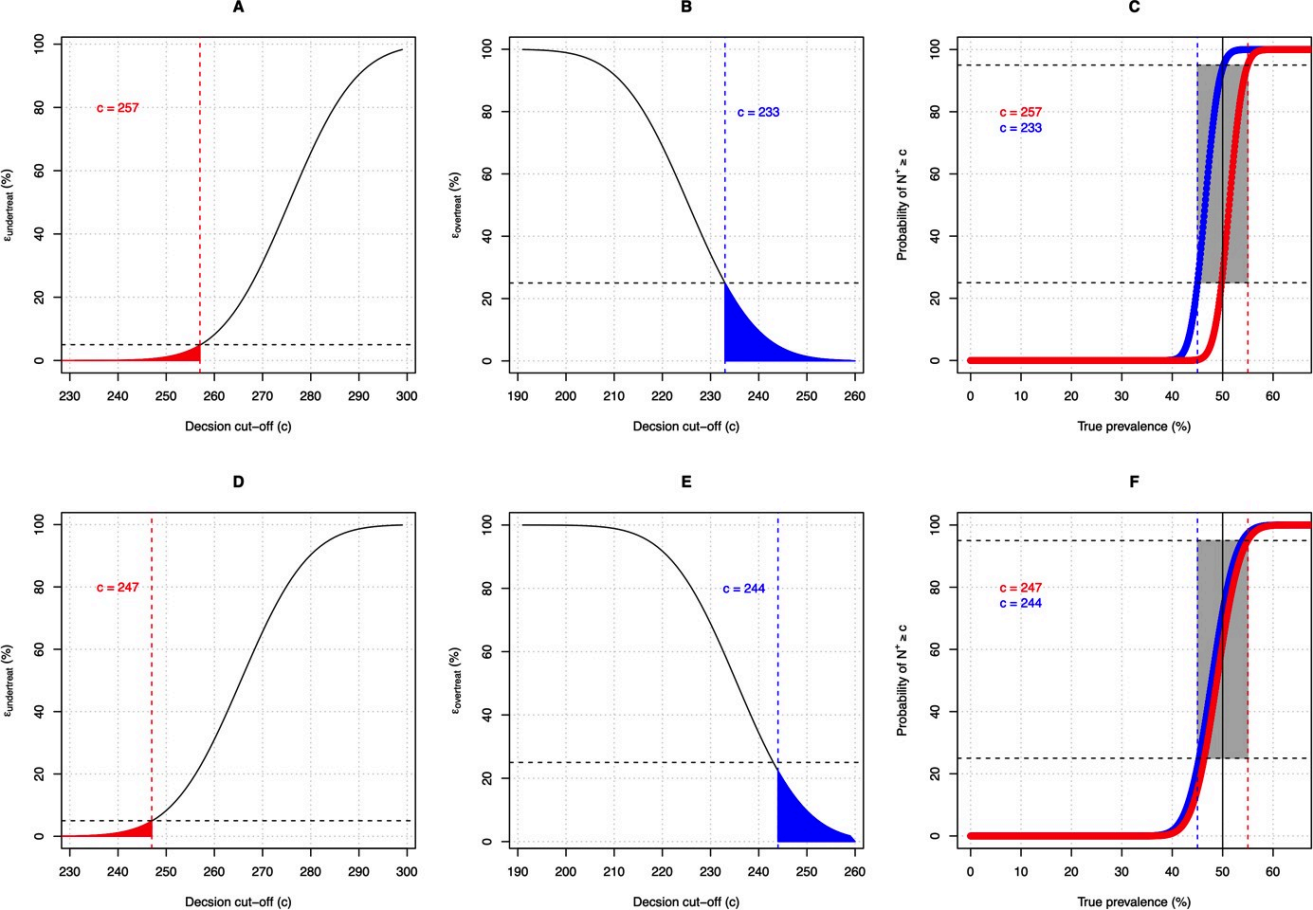
The process to determine the decision cut-off *c* in a LQAs framework. The different panels in this figure illustrate the process to determine the decision cut-off *c* when 500 subjects (*N*_*tot*_) are randomly recruited for both a perfect test (sensitivity (*Se*_*d*_) = specificity (*Sp*_*d*_) = 100%; **Panels A–C**) and an imperfect test (*Se*_*d*_ = *Sp*_*d*_ = 80%); **Panels D–F**). **Panels A** and **D** represent the cumulative error of prematurely reducing the preventive chemotherapy (PC) (*ε*_*undertreat*_) when the true underlying prevalence was arbitrarily set at 55% (*Prev*_*true*≥*T*_). The horizontal dashed line represents a *ε*_*undertreat*_ of 5%, the red dashed line represents the allowed possible decision cut-off *c* resulting in a *ε*_*undertreat*_≤5%. The red area under the curve highlight all possible values for *c* resulting in a *ε*_*undertreat*_≤5%. **Panels B** and **E** represent the cumulative error of selecting a PC frequency that is higher than needed (*ε*_*overtreat*_) when the true underlying prevalence was arbitrarily set at 45% (*Prev*_*true*<*T*_). The horizontal dashed line represents a *ε*_*overtreat*_ of 25%, the blue dashed line represents the lowest possible decision cut-off *c* resulting in a *ε*_*overtreat*_ of ≤ 25%. The blue area under the curve highlights all possible values for *c* resulting in a *ε*_*overtreat*_ of ≤ 25%. **Panels C** and **F** represent the probability (in %) of the number of positive test results (*N*^+^*)* in a random sample of *N*_*tot*_ subjects being at least *c* over a wide range of true underlying prevalence (*Prev*_*true*_) based on the two extreme decision cut-offs (red line: lowest possible value; blue line: highest possible value). The vertical straight line represents the program decision threshold *T* of 50%. The horizontal black dashed lines represent a *ε*_*overtreat*_ equal to 25% and a *ε*_*undertreat*_ equal to 5% (= 100% - 95%). The grey zone indicates the range of *Prev*_*true*_ for which decision making is inadequate (*ε*_*overtreat*_>25% (blue dashed line) and *ε*_*undertreat*_>5% (red dashed line). In this example, the grey zone ranges from 45% to 55% by design.

**Fig 2** further illustrates the process to determine the appropriate decision cut-off for two theoretical diagnostic tests. In this example, we determined the decision cut-off *c* for a sample size of 500 subjects (*N*_*tot*_) that allowed for *E*_*overtreat*_≤25% and *E*_*undertreat*_≤5% at a *Prev*_*true*<*T*_ arbitrarily set at 45% and at a *Prev*_*true*≥*T*_ arbitrarily set at 55% (program decision threshold *T* = 50%), respectively. To contrast the findings, we determined *c* for both a perfect (*Se*_*d*_ = *Sp*_*d*_ = 100%) and an imperfect test (*Se*_*d*_ = *Sp*_*d*_ = 80%).

For both theoretical diagnostic tests there is a range of possible values for *c*. For a perfect test (*Se*_*d*_ = *Sp*_*d*_ = 100%) any value between 233 (**Fig 2B**) and 257 (**Fig 2A**) can be used, whereas for an imperfect test (*Se*_*d*_ = *Sp*_*d*_ = 80%) the range of possible values is narrower, only ranging from 244 (**Fig 2E**) to 247 (**Fig 2D**). This reduction in options of *c* for an imperfect test is also reflected in panels representing the probability the number of positive test results (*N*^+^*)* in a random sample of *N*_*tot*_ subjects being at least *c* over a wide range of true underlying prevalence (*Prev*_*true*_) (**Fig 2C** and **2F**). Where both lines are almost overlapping for an imperfect test, there is a shift in *Prev*_*true*_ of 5-point percent between both lines for a perfect test.

#### Expansion of framework to MC-LQAS

**Fig 3:**
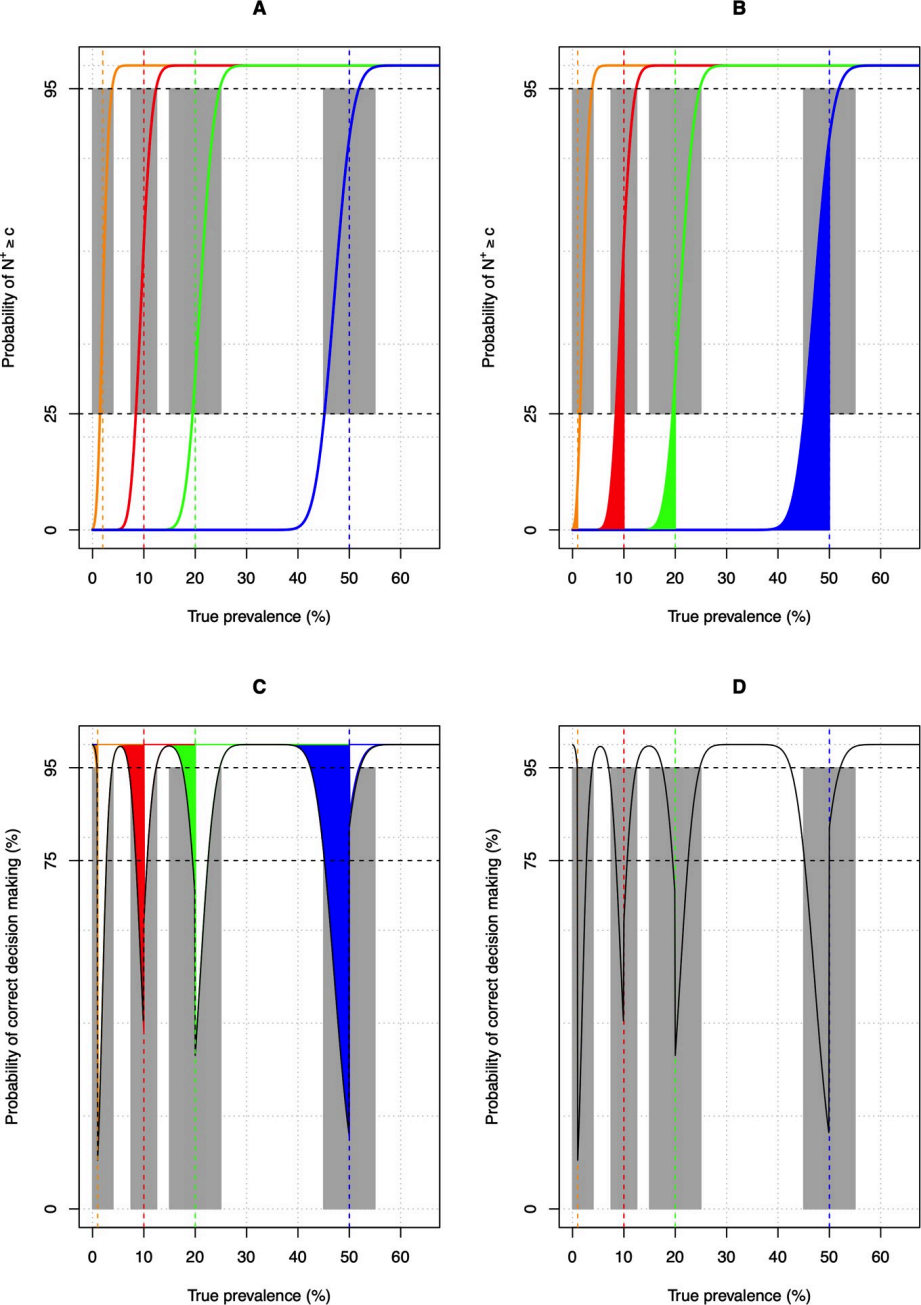
The build-up of multi-category LQAS for STH control program decision making using an imperfect test. The different panels illustrate the build-up of a multi-category LQAS around 4 program decision thresholds *T* (2%, 10%, 20% and 50%) when applying an imperfect test (sensitivity (*Se*_*d*_) = 76% and specificity (*Sp*_*d*_) = 99%) on 500 randomly selected subjects (*N*_*tot*_). **Panel A** provides the provides the probability (in %) of the number of positive test results (*N*^+^*)* in a random sample of *N*_*tot*_ subjects (= 500) being at least *c* separately for each of the 4 thresholds, their corresponding decision cut-offs (*c*_2%_ = 13, *c*_10%_ = 41, *c*_20%_ = 84, *c*_50%_ = 182) and true underlying prevalence *Prev*_*true*_ (*Prev*_*true<*2%_: 0.0%, *Prev*_*true≥*2%_: 4.0%; *Prev*_*true<*10%_: 7.5%, *Prev*_*true≥*10_: 12.5%; *Prev*_*true<*20%_: 15.0%, *Prev*_*true≥*20%_: 25.0%; *Prev*_*true<50*%_: 45.0%, *Prev*_*true≥*50_: 55.0%). Note that these *Prev*_*true*_-values define the borders of the grey zone around the program thresholds and for these *Prev*_*true*_-values *ε*_*overtreat*_≤25% and *ε*_*undertreat*_≤5%. The vertical straight line represents the program decision threshold *T* (orange: 2%, red: 10%, green: 20% and blue: 50%). The horizontal black dashed lines represent a *ε*_*overtreat*_ equal to 25% and a *ε*_*undertreat*_ equal to 5% (= 100% - 95%). The grey zone indicates the range of *Prev*_*true*_ for which decision making is inadequate (*ε*_*overtreat*_>25% and *ε*_*undertreat*_>5%). **Panel B** provides the same information as Panel A, but highlights the error of falsely scaling up the PC frequency (solid surfaces). **Panels C** and **D** represent the probability of correct program decision making across a wide range of *Prev*_*true*_, where **Panel C** provides an overview of the relative contribution of *ε*_*overtreat*_ (colored areas) in the program decision making.

In STH control programs decisions are made around multiple program decision thresholds, and hence a MC-LQAS (based on multiple decision cut-offs) would be more appropriate. In 2012, Olives et al. described the mathematical underpinnings of a multi-category LQAS for schistosomiasis based on 2 decision cut-offs, resulting in three categories (three-way MC-LQAS) [31]. **Fig 3** illustrates the built-up of a five-way MC-LQAS for program decisions around 4 program thresholds *T* currently used in STH programs (*T*_1_ = 2%, *T*_2_ = 10%, *T*_3_ = 20% and *T*_4_ = 50% [1]) when an imperfect test is used (*Se*_*d*_ = 76% and *Sp*_*d*_ = 98%; this combination of *Se*_*d*_ and *Sp*_*d*_ allowed for accurate decision making (see **Table 1**). **Fig 3A** provides the probability (in %) of the number of positive test results (*N*^+^*)* in a random sample of *N*_*tot*_ subjects (= 500) being at least *T*′ (see **(3)**) for each of the different thresholds, their corresponding decision cut-offs (*c*_2%_ = 13, *c*_10%_ = 41, *c*_20%_ = 84, *c*_50%_ = 182) and *Prev*_*true*_ (*Prev*_*true*<2%_: 0.0%, *Prev*_*true*≥2%_: 4.0%; *Prev*_*true*<10%_: 7.5%, *Prev*_*true*≥10_: 12.5%; *Prev*_*true*<20%_: 15.0%, *Prev*_*true*≥20%_: 25.0%; *Prev*_*true*<50%_: 45.0%, *Prev*_*true*≥50_: 55.0%). Note that these *Prev*_*true*_-values define the borders of the grey zone around the program thresholds and for these *Prev*_*true*_-values for which *ε*_*overtreat*_≤25% and *ε*_*undertreat*_≤5%. However, for a MC-LQAS we will need to consider the interaction between each of the 4 individual LQAS. For example, between 2 consecutive thresholds, there is not only the probability of prematurely reducing the PC frequency $\left(\varepsilon_{undertreat}=P(N^{+}<c_{T_{i}}|T_{i}\leq {Prev}_{true}<T_{i+1},{Se}_{d},{Sp}_{d},\;N_{tot})\right)$ there is also the probability of falsely scaling up the PC frequency $\left(\varepsilon_{overtreat}=P(N^{+}\geq c_{T_{i+1}}|T_{i}\leq {Prev}_{true}<T_{i+1},{Se}_{d},{Sp}_{d},\;N_{tot})\right)$. This *ε*_*overtreat*_ around each of the program thresholds is highlighted in **Fig 3B**. Combining both *ε*_*undertreat*_ and *ε*_*overtreat*_ results into the probability of making incorrect program decisions, or in other words 1−(*ε*_*undertreat*_+*ε*_*overtreat*_) or 1−*ε* provides the probability of correct program decision making. **Fig 3C** and **3D** represent the probability of correct program decision making across a wide range of *Prev*_*true*_, where **Fig 3C** provides an overview of the relative contribution of *ε*_*undertreat*_ and *ε*_*overtreat*_ in the program decision making. It is important to note that the different decision cut-offs $c_{T_{i}}$ in this example are not based on **(4)** and **(5)** for each threshold separately, rather they were determined using the equations below

$$P(N^{+}\geq c_{2\%}|{Prev}_{true<2\%},{Se}_{d},{Sp}_{d},\;N_{tot})\leq E_{1}$$ (6)

$$\begin{array}{c} P(c_{2\%}\leq N^{+}<c_{10\%}|{Prev}_{true\geq 2\%},{Se}_{d},{Sp}_{d},\;N_{tot})\leq 1-E_{2}\;\&\\ P(c_{2\%}\leq N^{+}<c_{10\%}|{Prev}_{true<10\%},{Se}_{d},{Sp}_{d},\;N_{tot})\leq 1-E_{3} \end{array}$$ (7)

$$\begin{array}{c} P(c_{10\%}\leq N^{+}<c_{20\%}|{Prev}_{true\geq 10\%},{Se}_{d},{Sp}_{d},\;N_{tot})\leq 1-E_{4}\;\&\\ P(c_{10\%}\leq N^{+}<c_{20\%}|{Prev}_{true<20\%},{Se}_{d},{Sp}_{d},\;N_{tot})\leq 1-E_{5} \end{array}$$ (8)

$$\begin{array}{c} P(c_{20\%}\leq N^{+}<c_{50\%}|{Prev}_{true\geq 20\%},{Se}_{d},{Sp}_{d},\;N_{tot})\leq 1-E_{6}\;\&\\ P(c_{20\%}\leq N^{+}<c_{50\%}|{Prev}_{true<50\%},{Se}_{d},{Sp}_{d},\;N_{tot})\leq 1-E_{7} \end{array}$$ (9)

$$P(N^{+}<c_{50\%}|{Prev}_{true\geq 50\%},{Se}_{d},{Sp}_{d},\;N_{tot})\leq E_{8}$$ (10)

where the *E* given *Prev*_*true*<*T*_ (indicated with the odd subscript) represents the allowed probability of selecting a PC frequency that is greater than indicated by the true underlying prevalence, and those *E* given *Prev*_*true*≥*T*_ (indicated with an even subscript) represents the allowed probability of prematurely reducing the PC frequency. In this example, the *E* given *Prev*_*true*<*T*_ was set at 25% and those given *Prev*_*true*≥*T*_ limit at 5%.

#### Determine sample size *N*_*tot*_ and decision cut−offs *c* for the required sensitivity and specificity within MC-LQAS

We will determine the sample size (*N*_*tot*_) and the corresponding decision cut-offs $c_{T_{i}}$ for those theoretical diagnostic tests that allowed for adequate or ideal program decision making. We varied the *N*_*tot*_ from 150–2,000 (by increments of 1), the corresponding decision cut-offs were based on **(6)–(10)**. In this MC-LQAS, we considered all thresholds currently used in STH control programs (2%, 10%, 20% and 50%). For the corresponding *Prev*_*true*_ limits, we used those used in the example illustrated in **Fig 3** (*Prev*_*true*<2%_: 0.0%, *Prev*_*true*≥2%_: 4.0%; *Prev*_*true<*10%_: 7.5%, *Prev*_*true*≥10_: 12.5%; *Prev*_*true*<20%_: 15.0%, *Prev*_*true*≥20%_: 25.0%; *Prev*_*true*<50%_: 45.0%, *Prev*_*true*≥50_: 55.0%). The *E* was set at 5% at *Prev*_*true*≥*T*_, *E* at *Prev*_*true*<*T*_ was either set at 25% for adequate program decision making and at 10% for ideal program decision making.

## Results

### Required sensitivity and specificity

**Fig 4:**
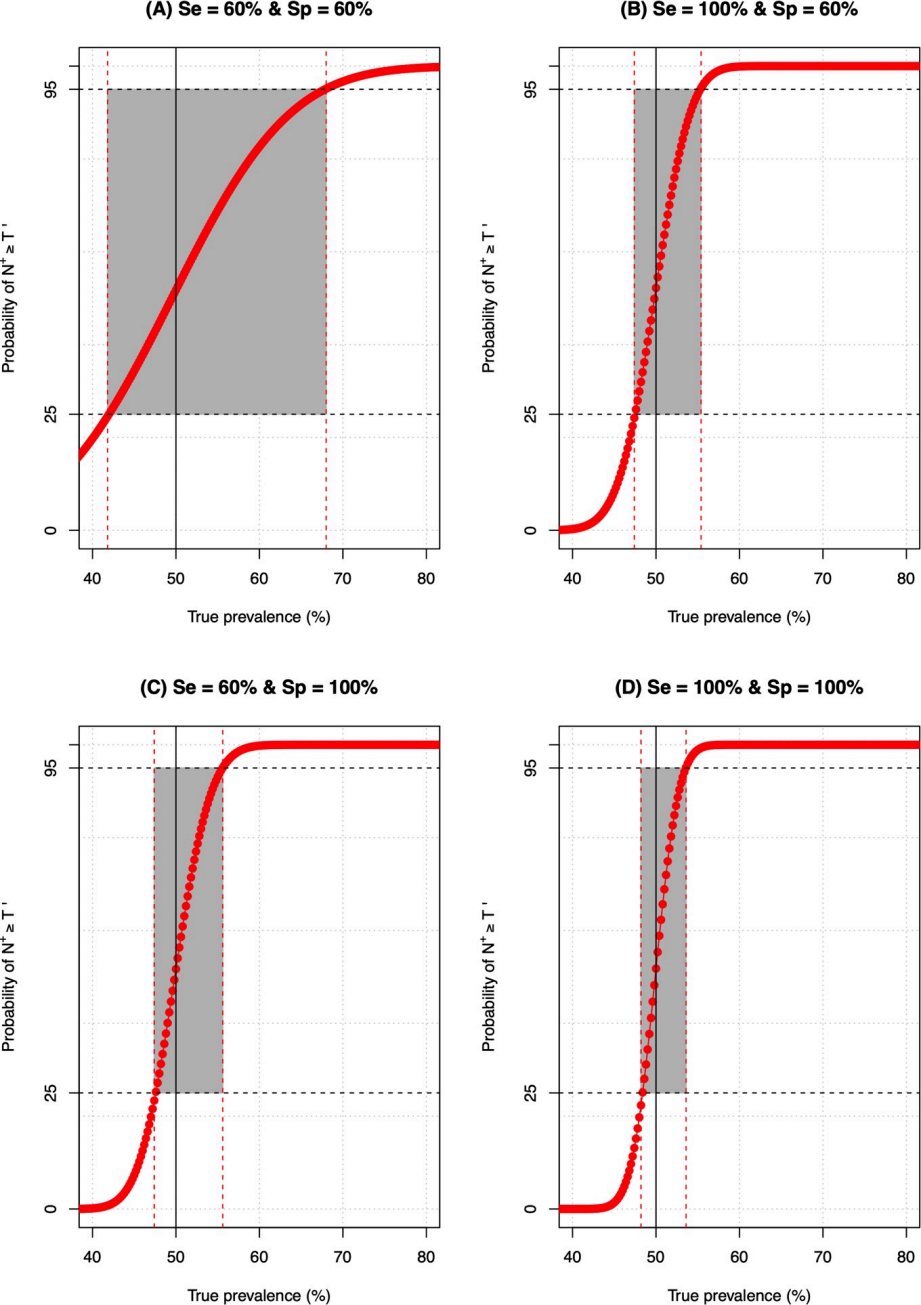
The program decision making around the 50% threshold for four theoretic diagnostic tests. The red line represents provides the probability (in %) of the number of positive test results (*N*^+^*)* in a random sample of *N*_*tot*_ subjects (= 500) being at least *T*′ (see **Eq 3**) using four theoretic distinct diagnostic tests (*D*_1_−*D*_4_). The diagnostic tests *D*_1_−*D*_3_ are imperfect diagnostic methods (**Panel A**: *Se*_*d*1_ = *Sp*_*d*1_ = 60%**; Panel B**: *Se*_*d*2_ = 100% and *Sp*_*d*2_ = 60%; **Panel C**: *Se*_*d*3_ = 60% and *Sp*_*d*3_ = 100%), whereas *D*_4_ is a perfect diagnostic method (**Panel D**: *Se*_*d*4_ = *Sp*_*d*4_ = 100%). The grey area represents the range of true underlying prevalence for which program decision is inadequate (*ε*_*overtreat*_>25% and *ε*_*undertreat*_>5%).

**Fig 5:**
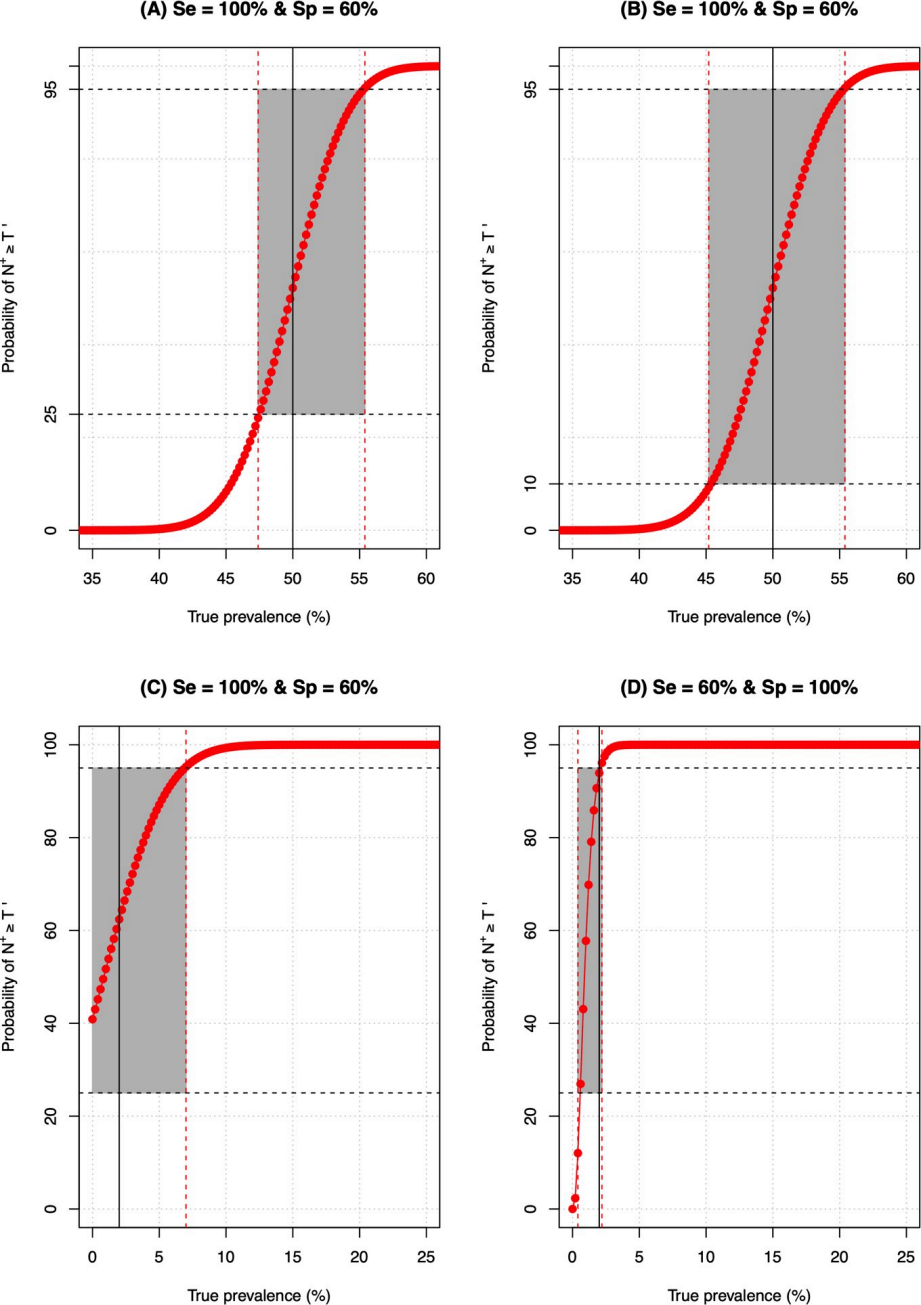
The impact of program decision errors and diagnostic performance on the grey zone. The red line in each panel represents the probability (in %) of the number of positive test results (*N*^+^*)* in a random sample of *N*_*tot*_ subjects (= 500) being at least *T*′ (see **Eq 3**) (**Panels A** and **B**: *T* = 50%, **Panels C** and **D**: *T* = 2%) using 2 theoretic distinct imperfect diagnostic tests *D*_1_ and *D*_2_ (*Se*_*d*1_ = 100% and *Sp*_*d*1_ = 60% (**Panels A**, **B** and **C**); *Se*_*d*2_ = 60% and *Sp*_*d*2_ = 100% (**Panel D**)). The grey area represents the range of true underlying prevalence for which program decision is inadequate (*ε*_*overtreat*_>25% and *ε*_*undertreat*_>5% (**Panels A, D** and **C**) or not ideal (*ε*_*overtreat*_>10% and *ε*_*undertreat*_>5% (**Panel B**).

**Figs 4** and **5** illustrate program decision making for a selection of the theoretic diagnostic tests, program decision thresholds and the level of reliable decision-making. **Fig 4** illustrates the program decision making for four theoretic distinct diagnostic tests (*D*_1_−*D*_4_) when decisions are made around the 50% program threshold. The diagnostic tests *D*_1_−*D*_3_ are imperfect diagnostic methods (**Fig 4A**: *Se*_*d*1_ = *Sp*_*d*1_ = 60%**; Fig 4B**: *Se*_*d*2_ = 100% and *Sp*_*d*2_ = 60%; **Fig 4C:** *Se*_*d*3_ = 60% and *Sp*_*d*3_ = 100%), whereas *D*_4_ is a perfect diagnostic method (**Fig 4D**: *Se*_*d*4_ = *Sp*_*d*4_ = 100%). **Fig 5** contrasts the impact of (i) program decision errors (*E*_*overtreat*_ = 25% (**Fig 5A**) *vs*. *E*_*overtreat*_ = 10% (**Fig 5B**)), (ii) program decision thresholds (50% (**Fig 5A**) *vs*. 2% (**Fig 5C**) and (iii) diagnostic performance (diagnostic test *D*_2_ (**Fig 5C**) *vs*. diagnostic test *D*_3_ (**Fig 5D**)) on the grey zone.

Taken together, these figures highlight three important aspects. First, they indicate that program decision making becomes inadequate (*ε*_*overtreat*_>25% and *ε*_*undertreat*_>5%) when the true underlying prevalence (*Prev*_*true*_) approaches the program decision threshold *T*, even if a perfect diagnostic method (*D*_4_) is applied. Second, they confirm that improved diagnostic tests (**Fig 4**), less stringent program errors (**Fig 5A** and **5B**) and lower program thresholds (**Fig 5B** and **5C**) allow for narrower grey zones. Third, it is important to note that improving the specificity has a greater impact on the program decision making than improving the sensitivity, and that the impact of specificity increases as the program decision threshold shifts to 2%. Indeed, for a program threshold of 50%, the grey zone of both diagnostic method *D*_2_ (*Se*_*d*2_ = 100% and *Sp*_*d*2_ = 60%) and *D*_3_ (*Se*_*d*3_ = 60% and *Sp*_*d*3_ = 100) are equally wide (**Fig 4**), whereas for program decision threshold of one percent, the grey zone of diagnostic method *D*_*3*_ is smaller compared to that one of diagnostic method *D*_2_ (2%: ~3-point percent *vs*. ~8-point percent) (**Fig 5C and 5D**).

**Fig 6:**
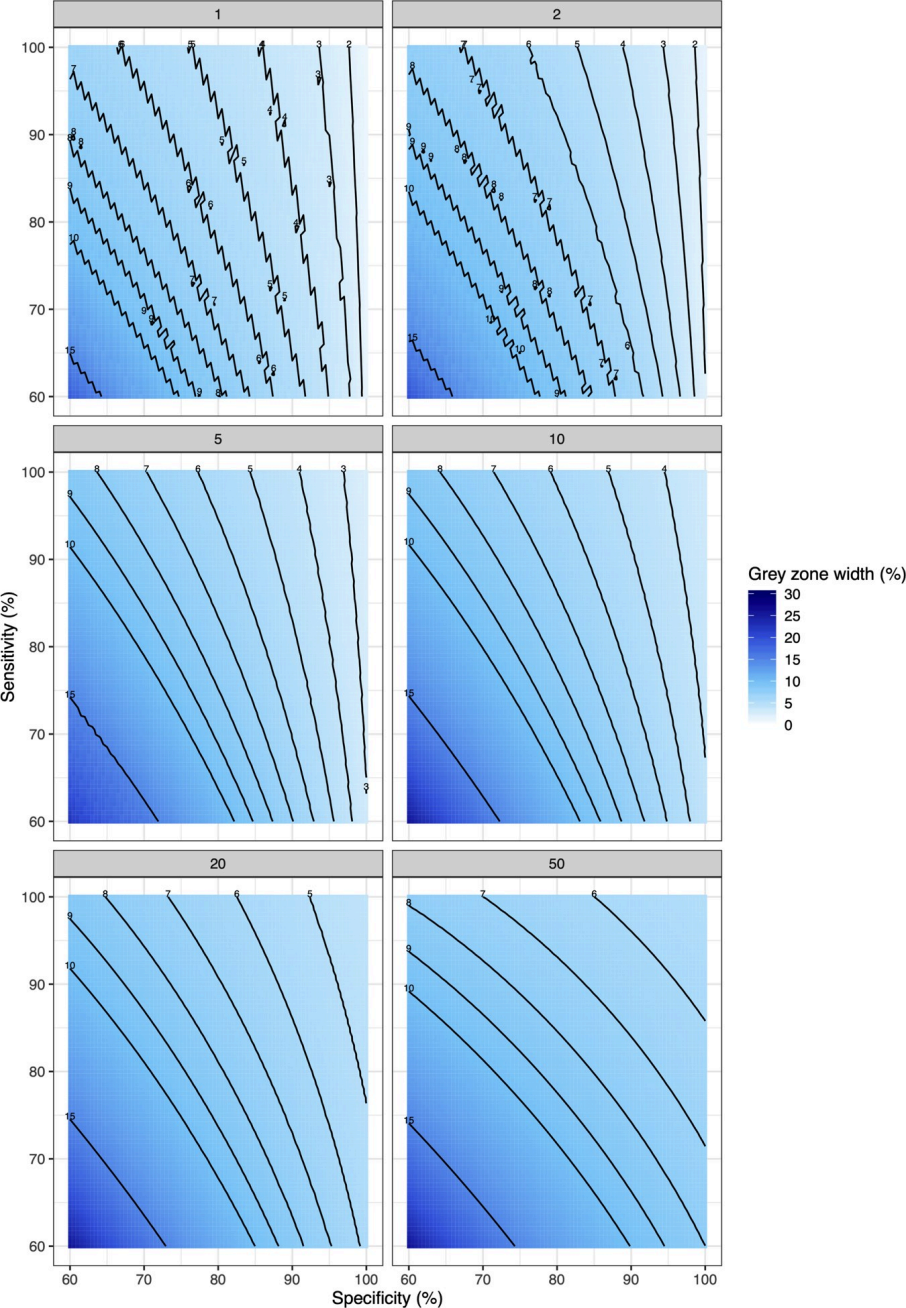
The width of grey zones around 6 program decision thresholds for 1,168 theoretic diagnostic tests. These contour plots illustrate the width of the grey zone for each of the unique combinations of sensitivity and specificity when decision making is adequate (*ε*_*overtreat*_≤25% and *ε*_*undertreat*_≤5%), each line representing the same width of grey zone. The number beside the line represents the floor value of the width of the grey zone in % (e.g., any value ≥10% and <11% is set at 10%).

**Fig 6** further summarizes the width of the grey zone for each of the 1,681 theoretic diagnostic tests by means of contour plots (each line represents the same width of grey zone) for adequate program decision making (**S1 Fig** provides the contour plots for ideal decision making). This figure highlights that multiple combinations of sensitivity and specificity can result in the same width of grey zone. For example, there are 408 combinations that result in a grey zone ~10-point percent wide around a program decision threshold *T* of 10%. However, for each of these combinations the sensitivity and specificity are inversely correlated (if sensitivity increases then the specificity decreases). Indeed, when the sensitivity is set at 60%, the specificity should not drop below ~83%. Similarly, a sensitivity of at least ~91% is required to obtain the same level of accurate decision making when the specificity is fixed at 60%. The figure also indicates that not all combinations can be recommended for monitoring and evaluating of STH programs, as the width of the grey zone would be too large to be relevant. An extreme case are the program decisions around a 2% threshold, where grey zones larger than 5-point percent would include a true underlying prevalence of zero, and hence would result in unnecessarily distributing drugs when disease has already been eliminated.

Of the 1,681 pairs of sensitivity (n = 41) and specificity (n = 41) that were evaluated, there were 207 combinations that allowed for adequate (*ε*_*overtreat*_≤25% and *ε*_*undertreat*_≤5%) program decision making and 61 that resulted in ideal program decisions (*ε*_*overtreat*_≤10% and *ε*_*undertreat*_≤5%) across each of the 6 program decision thresholds. In other words, they allowed for adequate or ideal decision making when the true underlying prevalence was zero and 100% across all thresholds. **Tables 1** and **2** provide an overview of the different possible diagnostic tests and their corresponding grey zone for *ε*_*overtreat*_ less or equal to 25% and 10% respectively. For simplicity, we have classified the width of the grey zone into three levels (1–3) for each threshold separately. The classification into these 3 levels was based for each program decision threshold separately on the 25^th^ and 75^th^ percentile of the width of the grey zones (level 1: width of grey zone < 25^th^ percentile; level 2: 75^th^ percentile > width of grey zone ≥ 25^th^ percentile; level 3: width of grey zone ≥ 75^th^ percentile (see **S1 Table**).

**Table 1:** The 207 diagnostic tests that allow for an adequate decision making. The table represents the width of the grey zone around the six program decision thresholds *T* (1%, 2%, 5%, 10%, 20% and 50%) that allowed for a sufficient decision making (*ε*_*overtreat*_≤25% and *ε*_*undertreat*_≤5%) for each of the 207 pairs of sensitivity (*Se*_*d*_) and specificity (*Sp*_*d*_). For simplicity, we have classified the width of the grey zone into three levels (1–3) for each threshold and *ε*_*undertreat*_ separately. This classification into 3 levels was based on the 25^th^ and 75^th^ percentile of the width of the grey zones (level 1: width of grey zone <25^th^ percentile; level 2: 75^th^ percentile > width of grey zone ≥ 25^th^ percentile; level 3: width of grey zone ≥ 75^th^ percentile (see **S1 Table**) across all potential diagnostic methods that allowed for adequate program decision making. In other words, each of these diagnostic methods allowed for adequate decision making (*ε*_*overtreat*_ is set at 25%) at a true underlying prevalence of zero. Diagnostic tests were considered ‘optimal’ (blue) when they resulted in level 1 grey zone in at least 3 out of the 6 thresholds and did not result in a level 3 grey zone in any of the 6 program thresholds. In all other cases, the diagnostic test was considered ‘minimal’ (white).

*Sp*_*d*_	*Se*_*d*_	Program thresholds (in %)						Type of test
		50	20	10	5	2	1	
100	74–100	1	1	1	1	1	1	Optimal
	63–73	2	1	1	1	1	1	Optimal
	60–62	2	1	1	1	1	2	Optimal
99	75–100	1	1	1	1	1	2	Optimal
	60–74	2	1	1	1	1	2	Optimal
98	76–100	1	1	1	1	1	2	Optimal
	69–75	2	1	1	1	1	2	Optimal
	67–68	2	1	1	1	1	3	Minimal
	66	2	1	1	1	2	3	Minimal
	64–65	2	1	1	1	1	3	Minimal
	62–63	2	2	1	1	1	3	Minimal
97	77–100	1	1	1	1	1	2	Optimal
	72–76	2	1	1	1	1	3	Minimal
	68–71	2	1	1	1	2	3	Minimal
	63–67	2	2	1	1	2	3	Minimal
96	92–100	1	1	1	1	1	2	Optimal
	84–91	1	1	1	1	1	3	Minimal
95	98–100	1	1	1	1	1	2	Optimal
	93–97	1	1	1	1	1	3	Minimal
	87–92	1	1	1	1	2	3	Minimal
	85–86	1	1	1	1	1	3	Minimal
94	96–100	1	1	1	1	1	3	Minimal
	86–95	1	1	1	1	2	3	Minimal

**Table 2:** The 61 diagnostic tests that allow for ideal decision making. The table represents the width of the grey zone around the six program decision thresholds *T* (1%, 2%, 5%, 10%, 20% and 50%) that allowed for a sufficient decision making (*ε*_*overtreat*_≤10% and *ε*_*undertreat*_≤5%) for each of the 61 pairs of sensitivity (*Se*_*d*_) and specificity (*Sp*_*d*_). For simplicity, we have classified the width of the grey zone into three levels (1–3) for each threshold separately. This classification into 3 levels was based on the 25^th^ and 75^th^ percentile of the width of the grey zones (level 1: width of grey zone < 25^th^ percentile; level 2: 75^th^ percentile > width of grey zone ≥ 25^th^ percentile; level 3: width of grey zone ≥ 75^th^ percentile (see **S1 Table**) across all potential diagnostic methods that allowed for adequate program decision making. In other words, each of these diagnostic methods allowed for adequate decision making (*ε*_*overtreat*_ is set at 25%) at a true underlying prevalence of zero). Diagnostic tests were considered ‘optimal’ (blue) when they resulted in level 1 grey zone around at least 3 out of the 6 thresholds and did not result in a level 3 grey zone in any of the 6 program thresholds. In all other cases, the diagnostic test was considered ‘minimal’ (white).

*Sp*_*d*_	*Se*_*d*_	Program thresholds (in %)						Type of test
		50	20	10	5	2	1	
100	96–100	1	1	1	1	1	1	Optimal
	81–95	2	1	1	1	1	1	Optimal
	70–80	2	2	1	1	1	1	Optimal
	61–69	2	2	1	1	1	2	Optimal
	60	3	2	1	1	1	2	Minimal
99	97–100	1	1	1	1	1	2	Optimal
	85–96	2	1	1	1	1	2	Optimal
	81–84	2	2	1	1	1	2	Optimal

Generally, each of these tables highlight four important aspects. First, they confirm that not all pairs of sensitivity and specificity allow for reliable decision making throughout all program phases. For example, combinations with specificity <94% are not included in **Table 1**. Second, they also confirm that diagnostic requirements become more stringent as program thresholds shift to 1%. This is because level 3 of the width of the grey zone in both tables is restricted by the program threshold of 1%. In other words, there are number of diagnostic tests that allowed for adequate or ideal program decision making around program decision thresholds between 2% and 50%, but failed to do so around a threshold *T* of 1%. Third, the requirements for both specificity and sensitivity are inversely correlated with each other; if the requirements are relaxed for one parameter, the requirements for the other one become more stringent for the other one. For example, if the specificity is 100% in **Table 1**, the lowest sensitivity to result in sufficient program decision making is 60%, whereas for a specificity of 94%, a sensitivity of at least 86% is required for sufficient decision making.

Fourth, when comparing **Table 1** and **Table 2** it becomes apparent that ideal program decisions require improved diagnostic tests. In contrast to an adequate program decision making (**Table 1**), for which there are 207 potential diagnostic tests, there are only 61 for ideal program decision making (**Table 2**). In addition, the requirements for specificity are more stringent. For an ideal decision making the specificity cannot drop below 99% (**Table 2**), whereas this was 94% across for an adequate decision making (**Table 1**).

In **Table 3** we cross tabulated the pairs of sensitivity and specificity across the two levels of program decision making (adequate *vs*. ideal) and two types of diagnostic test (minimal *vs*. optimal).

**Table 3:** The diagnostic performance of minimal and optimal diagnostic tests for adequate and ideal decision making. Diagnostic tests were considered ‘optimal’ when they resulted in level 1 grey zone in at least 3 out of the 6 thresholds and did not result in a level 3 grey zone in any of the 6 program thresholds. In all other cases, the diagnostic test was considered ‘minimal’. For simplicity, we have classified the width of the grey zone into three levels (1–3) for each threshold and *ε*_*undertreat*_ separately. The classification into these 3 levels was based on the 25^th^ and 75^th^ percentile of the width of the grey zones (level 1: width of grey zone < 25^th^ percentile; level 2: 75^th^ percentile > width of grey zone ≥ 25^th^ percentile; level 3: width of grey zone ≥ 75^th^ percentile (see **S1 Table**)). For an adequate decision making the *ε*_*overtreat*_≤25%, whereas for ideal decision making this *ε*_*overtreat*_≤10%. For both levels of decision making *ε*_*undertreat*_≤5%.

		Program decision making			
		Adequate		Ideal	
		Specificity	Sensitivity	Specificity	Sensitivity
**Type of test**	Minimal	98	62–68	100	60
		97	63–76		
		96	84–91		
		95	85–97		
		94	86–100		
	Optimal	100	≥ 60	100	≥ 61
		99	≥ 60	99	≥ 81
		98	≥ 69		
		97	≥ 77		
		96	≥ 92		
		95	≥ 98		

### Sample size and decision cut-offs for the required sensitivity and specificity

**Fig 7:**
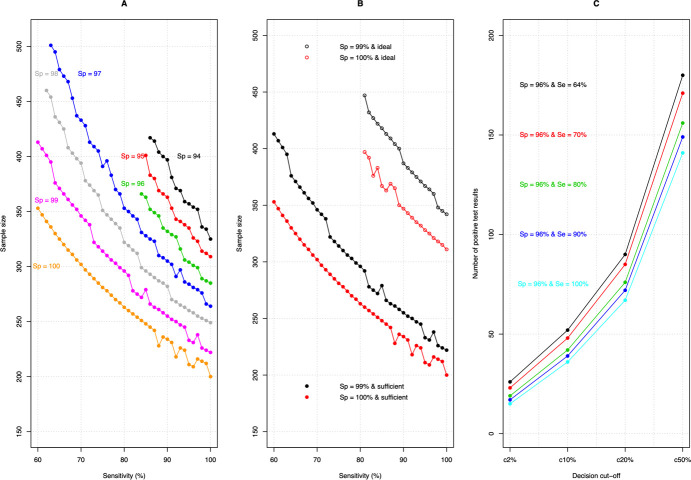
The variation in sample size and decision cut-off for a selection of the diagnostic tests. **Panel A** describes the variation in sample size across varying sensitivity (60–100%) and specificity (94–100%) when program decision making is adequate. **Panel B** contrasts the sample size between adequate and ideal program decisions for two diagnostic tests (specificity = 99% and specificity = 100%). **Panel C** illustrates the variation in decision thresholds (2%-50%) across four diagnostic tests with the same specificity (96%) but varying sensitivity (64%, 70%, 80% and 90%).

**Fig 7** summarizes the required sample size and the corresponding decision cut-offs *c*_*i*_ for the diagnostic tests summarized in **Table 3**. **Fig 7A** highlights that the required sample size decreases when the diagnostic performance improves. For example, where an imperfect diagnostic test (*Se*_*d*_ = *Sp*_*d*_ = 96%) requires 301 subjects, this is only 200 for a perfect test (*Se*_*d*_ = *Sp*_*d*_ = 100%). From the same panel we can deduce that improving the specificity has more impact on the sample size than improving sensitivity. For example, when improving the sensitivity from 96% to 100% when the specificity remains 96%, the sample size can only be reduced to 285, whereas improving the specificity from 96% to 100% when the sensitivity is fixed at 96%, the sample sizes can be further reduced to 209. Not unexpectedly, the sample size increases when an ideal rather than an adequate program decision making is required, and this is illustrated in **Fig 7B**. **Fig 7C** illustrates the variation in decision thresholds, highlighting that these values decrease when diagnostic tests become more perfect, which can be partially explained by the variation in sample size (see **Fig 7A**). The data used to determine the required diagnostic performance, the sample size and the corresponding decision cut-offs is provided **S1 Data**.

## Discussion

This study presents a generic and readily adaptable framework to explore the impact of diagnostic test sensitivity and specificity at the individual level on program decision making, in this instance applied to STH decision thresholds. Our results emphasize that specificity—rather than sensitivity—will become increasingly important at the end-game as decision-relevant prevalence thresholds become lower. Although it is commonly stated that sensitivity is the most important diagnostic parameter when the prevalence drops [32–34], our study suggests the opposite. Indeed, the outcome of the simulation study indicated that there are fewer options for specificity (≥94%) than for sensitivity (≥60%), when it comes to sufficient program decision making, and that increasing specificity improved the overall accuracy of program decision making (narrower grey zones; **Fig 6**, **Tables 1** and **2 and S1 Fig**). Expanding this to explore the outcome of decision-making using MC-LQAS further highlighted that improving specificity would result in significantly less operational costs in the field (fewer subjects required to make adequate or ideal program decisions (**Fig 7**)).

Generally, our findings are very much in line with recent similar work [28]. In fact, these observations are not unexpected, and this can be best illustrated by an extreme case. Assume the disease is truly absent in population and samples are processed with an imperfect diagnostic test, then the number of positive test results is determined by the specificity of the test only. For example, if we apply a test with a specificity of 95%, then there will be 5% (false) positive test results (**Eq 2**). Even if we have a true prevalence of 1% and a perfect sensitivity (100%), the majority of the positive tests will be false in nature.

### Sensitivity and specificity need to be determined for each program use case

In the present study, we focused on defining the required specificity and sensitivity that allowed for adequate/ideal decision-making at each program treatment threshold. This strategy will result in diagnostic tests that can be used across all program decision thresholds; however, there may be diagnostic tests that perform well at a single threshold that are excluded by this approach (e.g., tests that perform well in high-prevalence settings). Indeed, all combinations of sensitivity and specificity allow for adequate and ideal program decisions around program thresholds of 20% and 50%. In other words, the required diagnostic performance will need to be determined for each program use case separately (see also **Fig 6** and **S1 Fig**). For this, it will be equally important for the STH community to agree on the acceptable width of the grey zone separately for each program threshold, which in turn would provide a more justified criteria to classify diagnostic tests as ‘optimal’ and ‘minimal’ than those arbitrarily used in the present study.

### Specificity and sensitivity are inversely correlated

Although the lowest possible specificity and sensitivity is 94% and 60% for adequate decision making and 99% and 60% for ideal program decision making (**Table 3**), it is important to note that the diagnostic requirements for specificity and sensitivity are inversely correlated. As a consequence of this, it would be inappropriate to independently report the lowest values of specific and sensitivity into a TPP, as this would lead to the development of diagnostic tests that result in poor program decision making. Rather, combinations/pairs of specificity and sensitivity will need to be incorporated. **S2 Table** lists the pairs of sensitivity and specificity that were eventually recommended to the STH subgroup. They include the pairs summarized in **Table 3**, excluding all combinations with a perfect sensitivity or specificity, because this was deemed unrealistic.

### Currently used diagnostic methods may not allow for reliable decision making throughout an STH program

When comparing the recommended diagnostic performance (**S2 Table**) with the sensitivity and specificity for selection of currently available microscopic-based methods (e.g. direct smear, formol-ether concentration, Kato-Katz thick, McMaster, and (Mini-)FLOTAC) reported in a meta-analysis, it is clear that direct smear, formol-ether a single Kato-Katz and McMaster did not meet the requirements for detection of infections of any intensity for at least one of the three soil-transmitted helminths (Table 2 of [12]), and that in low endemic areas only FLOTAC would be a potential candidate (Table 3 of [12]). In a more recent study and assuming a perfect specificity [13], both a single and duplicate Kato-Katz, Mini-FLOTAC and qPCR did meet the required sensitivity for STH of any intensity (Table 3 of [13]), but when it concerns low intensity infections only qPCR remains as a potential candidate (Table 4 of [13]). FECPAK^G2^ did not meet any of the requirements. Although both studies indicate the potency of FLOTAC and qPCR, there are some important logistical obstacles to roll them out in large-scale deworming programs [16–18].

### Extension of the (MC)-LQAS framework allows to both develop and compare program decision algorithms for imperfect tests

To our knowledge this is the first description of a five-way MC-LQAS framework that accounts for imperfects test. The expansion of this framework not only allows for developing program decision algorithms across imperfect tests, but can also be used to gain insights into the operational cost. For example, we showed that additional investments to improve the test (e.g., the specificity) may provide downstream benefits of reducing the required survey sample sizes for making adequate programme decisions. This is because diagnostic tests with improved specificity require smaller sample sizes for the same level of program decision making. In other words, any additional cost per diagnostic test with improved diagnostic performance can be compensated by savings in operational costs for testing in the field or laboratory. Therefore, it is recommended to split up operational costs for testing into the material cost per test and the number of tests that can be processed in an hour by one person in future cost-analyses. This level of costing detail would lead to greater evidence-based recommendations in the TPPs.

### MC-LQAS framework needs to be adapted for 2-stage clustered sampling

In the current MC-LQAS framework we assumed that subjects are originating from the same cluster (e.g; community/school) and ignored the clustered nature of STH and assumed that these 500 subjects all represent one cluster (e.g. school/community). However, program decisions are not made at each cluster separately, rather decisions are made for a certain administrative or geographical area–the so-called implementation units–based on the aggregation of results across multiple clusters, with a number of subjects per cluster. In other words, programs employ 2-stage cluster sampling, whereby clusters are first chosen via random selection within an implementation unit and then a select number of subjects are chosen within each cluster. The development of a 2-stage cluster sampling MC-LQAS simulation approach was out of scope of the present study. A possible way forward would be to determine MC-LQAS around a 2-stage beta-binomial model, where the beta distribution describes the prevalence/proportion of positive test results across clusters and the binomial distribution the proportion of positive test results within a cluster.

### Both frameworks are generalizable to moderate-to-heavy intensity STH and any NTD program using population-based decision thresholds

Although the aforementioned frameworks were illustrated for program decision making around the prevalence of any STH infection, it is clear that both frameworks are agnostic to both the level of infection intensity and pathogen. For example, the results can also be used to make program decisions on whether the prevalence of moderate-to-heavy STH intensity infections has dropped below 2% [1]. Based on the diagnostic performance recommended in **S2 Table** and the recently reported probability of Mini-FLOTAC, McMaster and qPCR to correct classify moderate-to-heavy intensity infections when compared to Kato-Katz (Table 4 of [35]), we can deduce that only Mini-FLOTAC meets these requirements, though not for all STH species. Given that the schistosomiasis control programs use similar program decision thresholds [36], this framework will also provide insights for this NTD.

## Supporting information

S1 Table The thresholds to classify the width of the grey zone into three levels. This classification into 3 levels was based on the 25^th^ and 75^th^ percentile of the width of the grey zones across all potential diagnostic methods for each program threshold *T* separately that allowed for an adequate program decision making (level 1: width of grey zone < 25^th^ percentile; level 2: 75^th^ percentile > width of grey zone ≥ 25^th^ percentile; level 3: width of grey zone ≥ 75^th^ percentile).
(DOCX)

S2 Table The minimum and ideal sensitivity and specificity recommended by the STH subgroup. (DOCX)

S1 Fig The width of grey zones around 6 program decision thresholds for 1,168 theoretic diagnostic tests. These contour plots illustrate the width of the grey zone for each of the 1,168 unique combinations of sensitivity and specificity when decision making ideal (*ε*_*overtreat*_≤10% and *ε*_*undertreat*_≤5%)each line represents the same width of grey zone. The number of the beside the line represents the floor value of the width of the grey zone in % (e.g., any value ≥10% and <11% is set at 10%).
(TIF)

S1 Data The data used to determine the required diagnostic performance, the sample size and the corresponding decision cut-offs. (CSV)
