# Supplementary material for: Assessment of the required performance and the development of corresponding program decision rules for neglected tropical diseases diagnostic tests: Monitoring and evaluation of soil-transmitted helminthiasis control programs as a case study
Source: PLoS Negl Trop Dis. 2021 Sep 14;15(9):e0009740. doi: 10.1371/journal.pntd.0009740 (PMC8480900; doi:10.1371/journal.pntd.0009740)
Supplement: S1 Table — This classification into 3 levels was based on the 25th and 75th percentile of the width of the grey zones across all potential diagnostic methods for each program threshold T separately that allowed for an adequate program decision making (level 1: width of grey zone < 25th percentile; level 2: 75th percentile > width of grey zone ≥ 25th percentile; level 3: width of grey zone ≥ 75th percentile). (DOCX) [file pntd.0009740.s001.docx]

**Assessment of the required performance and the development of corresponding program decision rules for neglected tropical diseases diagnostic tests: monitoring and evaluation of soil-transmitted helminthiasis control programs as a case study**

Bruno Levecke^1#^, Luc E. Coffeng^2^, Christopher Hanna^3^, Rachel L Pullan^4^, Katherine Gass^5^

^1^Department of Virology, Parasitology, Immunology and Physiology, Ghent University, Merelbeke, Belgium

^2^Department of Public Health, Erasmus MC, University Medical Centre Rotterdam, Rotterdam, the Netherlands

^3^Global Project Partners, LLC, Oakland, CA, USA

^4^Department of Disease Control, London School of Hygiene and Tropical Medicine, London, UK

^5^Neglected Tropical Diseases Support Centre, The Task Force for Global Health, Decatur, USA

**Running title:** Required diagnostic performance for NTDs tests

^#^Corresponding author: bruno.levecke@ugent.be

**S1 Table. The thresholds to classify the width of the grey zone into three levels.** This classification into 3 levels was based on the 25^th^ and 75^th^ percentile of the width of the grey zones across all potential diagnostic methods for each program threshold *T* separately that allowed for an adequate program decision making (level 1: width of grey zone <25^th^ percentile; level 2: 75^th^ percentile> width of grey zone $\geq$25^th^ percentile; level 3: width of grey zone $\geq$75^th^ percentile).

| **Program decision threshold (%)** | **25^th^ percentile width of the grey zone (point-%)** | **75^th^ percentile width of the grey zone (point-%)** |
| --- | --- | --- |
| 1% | 1.8% | 3.0% |
| 2% | 3.4% | 5.6% |
| 5% | 5.2% | 9.6% |
| 10% | 5.8% | 10.2% |
| 20% | 6.4% | 10.4% |
| 50% | 7.2% | 10.8% |
