# Supplementary material for: Assessment of the required performance and the development of corresponding program decision rules for neglected tropical diseases diagnostic tests: Monitoring and evaluation of soil-transmitted helminthiasis control programs as a case study
Source: PLoS Negl Trop Dis. 2021 Sep 14;15(9):e0009740. doi: 10.1371/journal.pntd.0009740 (PMC8480900; doi:10.1371/journal.pntd.0009740)
Supplement: S2 Table — (DOCX) [file pntd.0009740.s002.docx]

**Assessment of the required performance and the development of corresponding program decision rules for neglected tropical diseases diagnostic tests: monitoring and evaluation of soil-transmitted helminthiasis control programs as a case study**

Bruno Levecke^1#^, Luc E. Coffeng^2^, Christopher Hanna^3^, Rachel L Pullan^4^, Katherine Gass^5^

^1^Department of Virology, Parasitology, Immunology and Physiology, Ghent University, Merelbeke, Belgium

^2^Department of Public Health, Erasmus MC, University Medical Centre Rotterdam, Rotterdam, the Netherlands

^3^Global Project Partners, LLC, Oakland, CA, USA

^4^Department of Disease Control, London School of Hygiene and Tropical Medicine, London, UK

^5^Neglected Tropical Diseases Support Centre, The Task Force for Global Health, Decatur, USA

**Running title:** Required diagnostic performance for NTDs tests

^#^Corresponding author: bruno.levecke@ugent.be

**S2 Table. The minimum and ideal sensitivity and specificity recommended by the STH subgroup.**

| **Minimum** |  |  | **Ideal** |  |
| --- | --- | --- | --- | --- |
| Specificity (%) | Sensitivity (%) |  | Specificity (%) | Sensitivity (%) |
| 99 | $\geq$60 |  | 99 | $\geq$60 |
| 98 | $\geq$62 |  | 98 | $\geq$69 |
| 97 | $\geq$63 |  | 97 | $\geq$77 |
| 96 | $\geq$84 |  | 96 | $\geq$92 |
| 95 | $\geq$85 |  | 95 | $\geq$98 |
| 94 | $\geq$86 |  |  |  |
