# Supplementary figures and images for: Assessment of the required performance and the development of corresponding program decision rules for neglected tropical diseases diagnostic tests: Monitoring and evaluation of soil-transmitted helminthiasis control programs as a case study
Source: PLoS Negl Trop Dis. 2021 Sep 14;15(9):e0009740. doi: 10.1371/journal.pntd.0009740 (PMC8480900; doi:10.1371/journal.pntd.0009740)

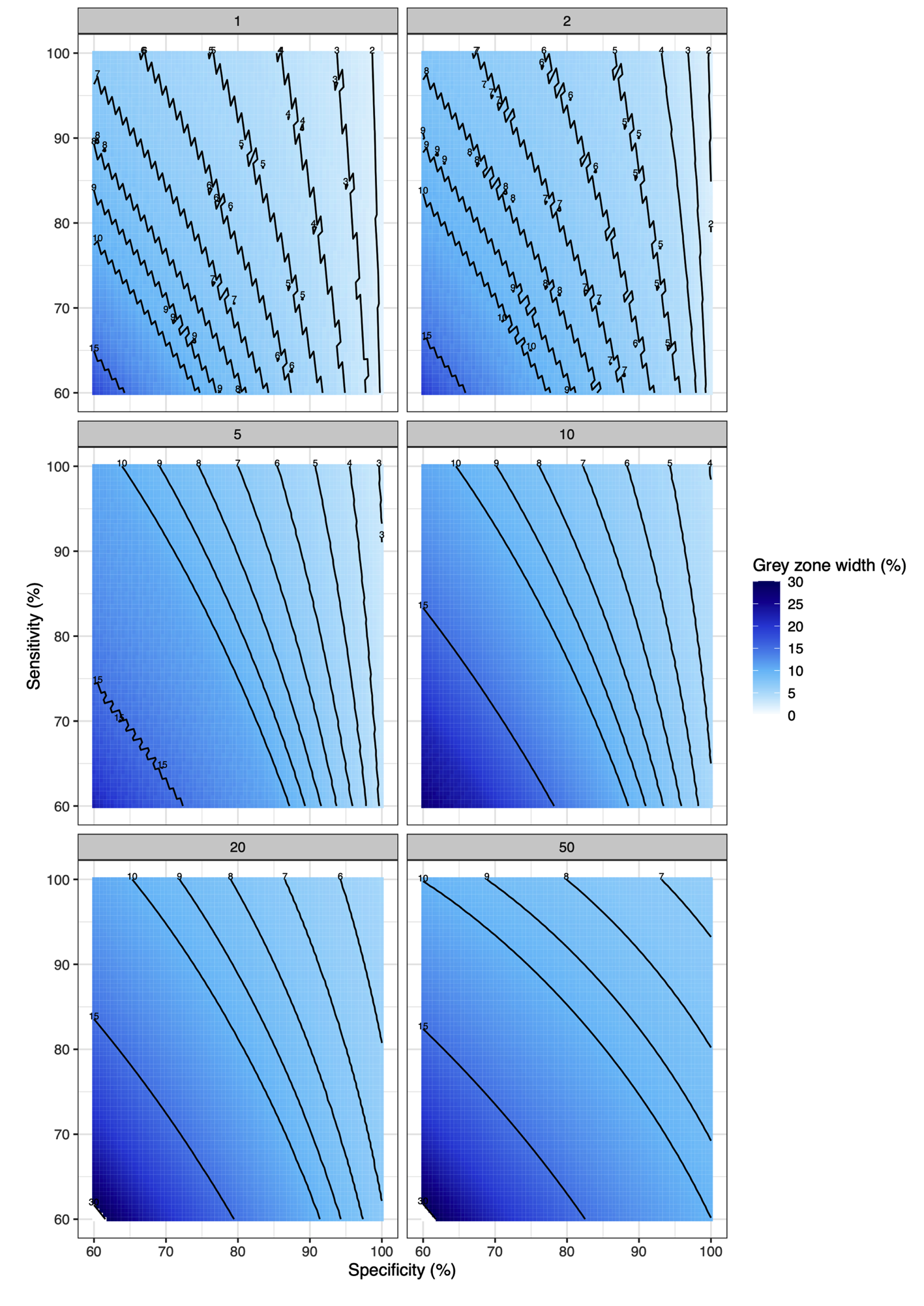

Supplement: S1 Fig — These contour plots illustrate the width of the grey zone for each of the 1,168 unique combinations of sensitivity and specificity when decision making ideal (εovertreat≤10% and εundertreat≤5%)each line represents the same width of grey zone. The number of the beside the line represents the floor value of the width of the grey zone in % (e.g., any value ≥10% and <11% is set at 10%). (TIF) [file pntd.0009740.s003.tif]
